# Concerns for labor analgesia and autism spectrum disorders

**DOI:** 10.1007/s00540-020-02880-x

**Published:** 2020-11-24

**Authors:** Manabu Saito, Kazuhiko Nakamura, Kazuyoshi Hirota

**Affiliations:** 1 Department of Neuropsychiatry, Graduate School of Medicine, Hirosaki University, 5 Zaifu-cho, Hirosaki, 036-8562 Japan; 2 Department of Anesthesiology, Graduate School of Medicine, Hirosaki University, 5 Zaifu-cho, Hirosaki, 036-8562 Japan

**Keywords:** Labor epidural analgesia, Autism spectrum disorders, Hazard ratio

To the Editor:

We read with great interest the Association between Epidural Analgesia during Labor and Risk of Autism Spectrum Disorders in Offspring by Chunyuan Qiu et al. published in JAMA Pediatrics on October 12, 2020 [1]. The findings were that in this multiethnic population-based clinical birth cohort that included 147,895 children, Autism spectrum disorders (ASD) were diagnosed in 1.9% of the children delivered vaginally with labor epidural analgesia (LEA) vs. 1.3% without the exposure, 37% relative increase in risk that was significant after adjusting for potential confounders. We appreciate the authors’ efforts, however, we have some concerns in this study as ASD specialists [2].

First of all, they used Cox proportional hazards regression analysis to estimate the hazard ratio (HR) of ASD associated with LEA exposure, but as described in the limitation, it cannot be interpreted as a causal relationship. Because they do not consider other risk factors in previous reports as covariates that affect outcomes. For example, the information of ASD diagnosis in parents is needed given that autism is currently estimated to be 40–80% genetic. In addition, information such as viral infections or exposure to air pollution during pregnancy and perinatal hypoxic encephalopathy should be needed as non-genetic factors of ASD. They should show that LEA exposure is one of the hundreds of environmental factors for ASD.

Second, the main outcome is the clinical diagnosis of ASD, but the basis for the diagnosis is unclear and no other comorbidities (such as ADHD) have been identified, it cannot be determined whether the outcome ASD diagnosis is reliable. In addition, the sample ratio is 3:1, and the effect of the number of samples on detection power cannot be denied. Further verification is needed on the association between LEA and ASD. Therefore, the hazard rate associated with LEA could be an error.

The final concern is the impact of this title on mothers who choose LEA. Some of the mothers may feel anxious and guilty. Although it is clearly stated in the paper that there is no causal relationship, it is difficult to read that point from the title and abstract. We strongly hope that many people will be able to accurately understand the interpretation of this research result as LEA is getting popular in Japan [3].
